# Supplementary material for: Oxygen microenvironments in Escherichia coli biofilm nutrient transport channels: insights from complementary sensing approaches
Source: Microbiology (Reading). 2025 May 6;171(5):001543. doi: 10.1099/mic.0.001543 (PMC12056250; doi:10.1099/mic.0.001543)
Supplement: Uncited Supplementary Material 1. [file mic-171-01543-s001.pdf]

Supplementary Information for '*Oxygen Microenvironments in E. coli Biofilm Nutrient Transport Channels: Insights from Complementary Sensing Approaches*'

1 Supplementary Material

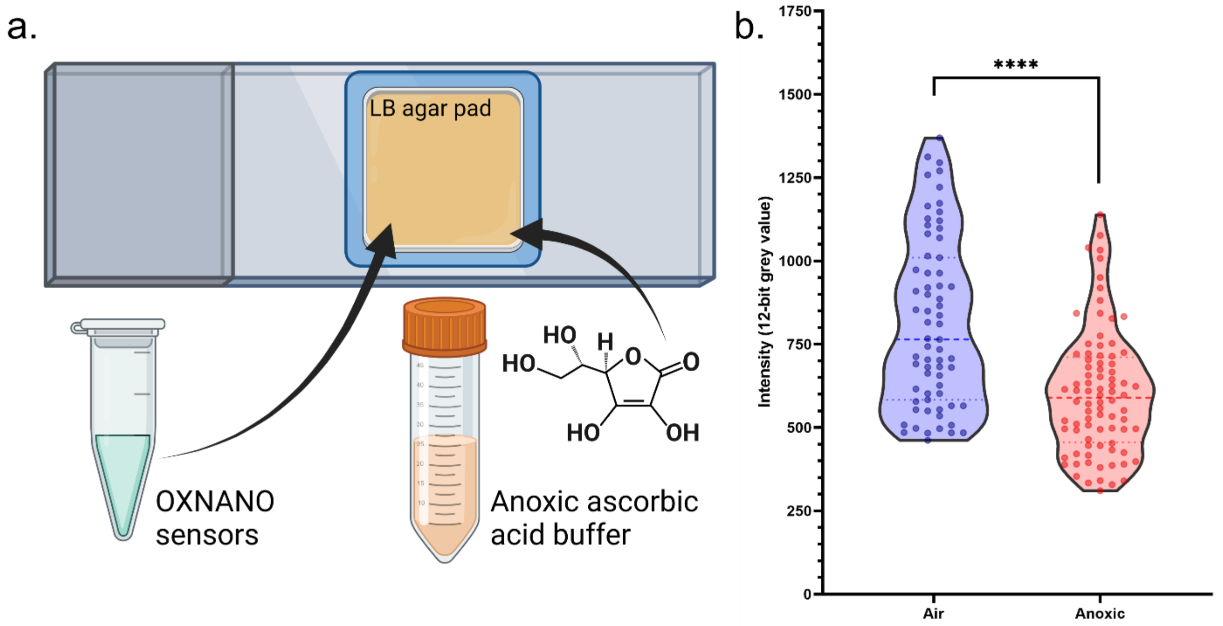

Supplementary Figure 1. Oxygen Nanosensor Range Validation. (a) An experimental schematic to determine the difference in oxygen nanosensor emission intensity under atmospheric and anoxic conditions. Ascorbic acid buffer acts as a strong reducing agent and sequesters molecular oxygen rapidly once the agar pad is sealed with a coverglass. Atmospheric oxygen conditions were achieved by imaging the exposed pad containing a lawn of oxygen nanosensors **(b)** The fluorescence emission intensity of oxygen nanosensors was compared under atmospheric and anoxic conditions using a confocal laser scanning microscope. The emission intensity of beads from six replicate slides was compared, with median atmospheric intensity of 766 intensity units (IQR = 425) and median anoxic intensity of 598 intensity units (IQR = 255). Statistical significance was calculated using a Mann-Whitney test ($P < 0.01$, ***) ($N_{External}$ = 68, $N_{Internal}$ = 87; acquired over 6 experimental replicates).

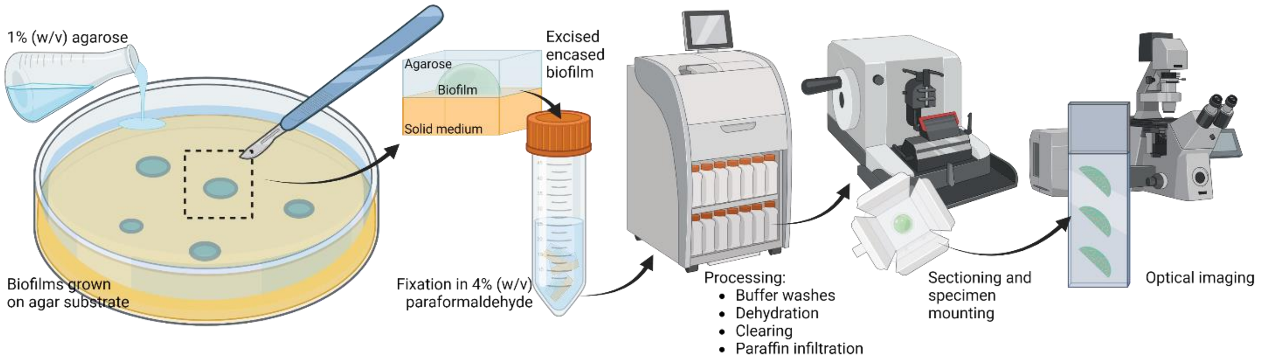

Supplementary Figure 2. Methodology for colony biofilm thin sectioning. A diagrammatic workflow for agarose stabilisation, fixation, paraffin embedding and thin sectioning of *E. coli* macrocolony biofilms. Specimens are embedded in cooled molten agarose, excised from a Petri dish and fixed in 4% (w/v) paraformaldehyde. Fixed blocks are then processed and paraffin embedded before microtome sectioning and mounting for optical imaging.

Strain/Plasmid ID	Genotype	Source
JM105 (DSMZ-3949)	Wild type<br>*endA1 glnV44 sbcB15 rpsL thi-1* Δ(*lac-proAB*) [F' *traD36 proAB*$^+$ *lacI*^{q} *lacZ*Δ*M15*] *hsdR4*($r_K^- m_K^+$)	DSMZ, Germany
JM105 miniTn7::*HcRed*	Gm^R P.$_{A1/04/03}$::*HcRed*	[66]
JM105 miniTn7::*gfp*	Gm^R P.$_{A1/04/03}$::*gfp*	[66]
NEB-5alpha	*fhuA2*Δ(*argF-lacZ*)*U169 phoA glnV44* Φ*80*Δ(*lacZ*)*M15 gyrA96 recA1 relA1 endA1 thi-1 hsdR17*	New England Biolabs, USA
pAW9	Oxygen reporter plasmid<br>Cm^R P$_{cco2}$::*gfp*	[28]

Supplementary Table 1. List of bacterial strains and plasmids.
